# Differences in the Processing of Chinese Transitive and Intransitive Verbs at the Behavioral Response and Neural Activity Levels

**DOI:** 10.3390/bs16030334

**Published:** 2026-02-27

**Authors:** Xin Wang, Dandan Liang, Yiming Yang

**Affiliations:** 1 School of Humanities and Arts, China University of Mining and Technology, Xuzhou 221116, China; super_wangxin2010@163.com; 2 School of Liberal Arts, Nanjing Normal University, Nanjing 210097, China; 3 School of Linguistic Sciences and Arts, Jiangsu Normal University, Xuzhou 221009, China; yangym@jsnu.edu.cn

**Keywords:** Chinese, intransitive verb, transitive verb, accuracy rate, reaction time, bilateral angular gyri, left supramarginal gyrus, left inferior frontal gyrus

## Abstract

In Chinese, intransitive verbs can take direct objects in certain constructions, and transitive verbs can also be used without objects. These characteristics have long sparked debates about whether verbs can be divided into intransitive and transitive verbs in Chinese. Using E-Prime software (3.0 version) and functional magnetic resonance imaging (fMRI) technology, we investigated the behavioral responses and neural activities of native speakers when processing Chinese intransitive and transitive verbs. Behavioral data showed that the accuracy rate for Chinese intransitive verbs was significantly higher than that for transitive verbs, while the reaction time was significantly shorter. fMRI data revealed that compared with Chinese intransitive verbs, transitive verbs elicited significantly stronger activation in brain regions such as the bilateral angular gyri (BA39), left supramarginal gyrus (BA40), and left inferior frontal gyrus (BA44). The bilateral angular gyri and left supramarginal gyrus may be associated with more intricate argument semantic representation of the Chinese transitive verb, while the left inferior frontal gyrus may reflect their more complex syntactic structure representation. The above experimental results indicate that processing Chinese transitive verbs requires greater cognitive effort and involves more complex neural activities compared to intransitive verbs, which demonstrates that verbs in Chinese should be subdivided into intransitive and transitive verbs.

## 1. Introduction

Verbs are generally classified into intransitive and transitive verbs based on their ability to take direct objects—intransitive verbs cannot take direct objects, while transitive verbs must take direct objects (X. Wang, 2023; X. Wang et al., 2021). For example, 1a–1c show that the intransitive verb “fly” in English can only take a subject. If an object is to be added, the preposition “to” must be placed before it. On the other hand, 2a–2c indicate that the transitive verb “invite” in English must take an object. Even if the object has been mentioned in the preceding context, the transitive verb must still be followed by a pronoun referring to that object. The asterisk before the example means the sentence is incorrect.
1a The bird flies.2a The student invites a teacher.1b * The bird flies the park.2b * The student invites.1c The bird flies to the park.2c There is a teacher in the room, and the student invites him.

A series of theoretical and empirical research has demonstrated that verbs can be categorized into intransitive verbs and transitive verbs. From the perspective of theoretical research, the lexicalist approach (Horvath & Siloni, 2011; Meltzer-Asscher et al., 2013) posits that the lexical information of an intransitive verb specifies that it takes one obligatory argument, which is assigned a specific thematic role (e.g., agent). By contrast, the lexical information of a transitive verb dictates that it selects two obligatory arguments, each of which is assigned a distinct thematic role (e.g., agent and patient). The syntactic structure of a transitive verb undergoes two instances of merge: first, the object merges with the transitive verb; second, the subject merges with the verb phrase formed by the transitive verb and the object. In contrast, the syntactic structure of an intransitive verb only involves one instance of merge, in which the subject merges with the intransitive verb. Semantically, transitive verbs involve the semantic representation of a subject and an object, whereas intransitive verbs only involve the semantic representation of a subject. It should be noted that transitivity initially focused on the semantic differences between verbs. With the advancement of theoretical research, studies have revealed that transitivity features also exhibit syntactic variations. Similarly, valency first centered on the differences in the syntactic structures of verbs and later extended its focus to semantic differences (X. Wang, 2023; Zhou, 2024).

Additionally, empirical research has investigated the processing of intransitive and transitive verbs. The findings reveal that transitive verbs entail more complex processing than their intransitive counterparts. The behavioral experiments adopted reaction time and accuracy rate as the primary indicators to investigate the processing of intransitive verbs and transitive verbs among healthy participants, as well as patient groups such as those with aphasia and Alzheimer’s disease. The results of these studies indicated that participants exhibited longer reaction times and lower accuracy rates when processing transitive verbs compared with intransitive verbs (Kim & Thompson, 2000, 2004; Thompson et al., 1997, 2010). In turn, several neurocognitive experiments utilized fMRI to investigate the neural activity associated with the processing of intransitive and transitive verbs, and found that the brain activation patterns of the latter were more complex than those of the former (Den Ouden et al., 2009; Meltzer-Asscher et al., 2015; Thompson et al., 2007, 2013). These fMRI studies suggest that the angular gyrus, supramarginal gyrus, and temporoparietal regions are associated with the more complex semantic representations of transitive verbs, while the left inferior frontal gyrus reflects the more complex syntactic representations of transitive verbs.

In addition, whether Chinese verbs can be divided into intransitive and transitive verbs has long been debated. Some studies argue that the lexical information of Chinese verbs also encodes transitivity features (Jin & Yu, 2022; W. Wang & Chen, 2017; X. Wang, 2023; X. Wang & Zhou, 2023; Xu, 2001). These studies have further examined the syntactic and semantic differences between Chinese intransitive and transitive verbs. Syntactically, transitive verbs can be embedded in passive constructions and bǎ-constructions, whereas intransitive verbs cannot be incorporated into either of these two structures. Examples 3a–3c demonstrate that the transitive verb “打” (to hit) in Chinese can form an active structure with the S-V-O word order, as well as passive constructions and bǎ-constructions. In contrast, Examples 4a–4c show that the intransitive verb “哭” (to cry) in Chinese can only form an active structure with the S-V word order, and cannot be used in passive constructions or bǎ-constructions. In the Chinese bǎ-construction, the object of a transitive verb follows the preposition bǎ and precedes the verb, emphasizing the disposal state of the object. Semantically, transitive verbs in Chinese have a direct semantic association with both of their two obligatory arguments, whereas intransitive verbs in Chinese have a direct semantic association only with their single obligatory argument. For example, in Example 3a, the subject “小明” (Xiǎomíng) acts as the agent of the transitive verb “打” (to hit), while the object “小红” (Xiǎohóng) functions as its patient. In contrast, in Example 4a, only the subject “小红” has a direct semantic relationship with the intransitive verb “哭” (to cry), serving as the agent of the action.
3a.小明打了小红。(transitive active construction)
Xiǎomínghit-perfXiǎohóng
‘ Xiǎomíng hit Xiǎohóng.3b.小红被 (小明)打了。(passive construction)
Xiǎohóngwas (by Xiǎomíng)hit-perf
‘ Xiǎohóng was hit (by Xiǎomíng)3c.小明把 小红打了。(bǎ-construction)
Xiǎomíngbǎ Xiǎohónghit-perf
‘ Xiǎomíng hit Xiǎohóng.4a.小红哭了。(intransitive active construction)
Xiǎohóngcry-perf
‘ *Xiǎohóng cried.4b.*小红被哭了。(passive construction)
Xiǎohóngwas cried-perf
‘ *Xiǎohóng was cried.4c.*小红把哭了。(bǎ-construction)
Xiǎohóngbǎcry-perf
‘ *Xiǎohóng bǎ cried.

Other studies point out that intransitive verbs in Chinese can also directly take nominal elements as objects, thus exhibiting transitive usages. Conversely, transitive verbs in Chinese can also appear without objects, thereby manifesting intransitive usages (Chen, 2018; Z. Liu, 2007; Shen, 2018; Wu & Li, 2014). In Example 5a, the verb “飞” (to fly) takes only a subject, which is an intransitive usage; in Example 5b, however, the verb “飞” takes a subject and also directly takes a locative object. In Example 5c, the verb “邀请” (to invite) takes both a subject and an object, which is a transitive usage; in Example 5d, by contrast, the verb “邀请” takes only a subject without an object following it. It should be pointed out that many high-frequency verbs in English can also occur without an object, with the typical generic object implied (e.g., *I want to eat/read*). The phenomenon of typical generic object omission also exists in Chinese (e.g., 你吃了吗？/Have you eaten?). What is different from English is that, in addition to a limited number of typical generic object omissions, Chinese also has a large number of instances where objects are omitted based on the preceding or following context (see Example 5d).
5a晚上燕子飞了。


eveningswallowfly-perf


‘ The swallow flew away in the evening5b明天小红飞 北京。

TomorrowXiǎohóngflyBeijing

‘ Xiǎohóng will fly to Beijing tomorrow5c昨天学生邀请了老师。

Yesterdaystudentinvite-perfteacher

‘ The students invited the teacher yesterday5d昨天小明已经邀请了老师，
YesterdayXiaomingalreadyinvite-perfteacher
我今天不 会邀请了。
Itodaynotwillinvite-model particle
‘ Xiǎomíng had already invited the teacher yesterday,
so I won’t invite him today

In response to the above-mentioned view, related studies point out that Example 5b actually expresses the meaning of “明天小红飞**往 (to)** 北京”. This type of construction is a preposition–ellipsis construction (Guo, 1999; X. Liu, 2011; X. Wang, 2023; X. Wang & Zhou, 2023; B. Zhang, 2019). The locative element following the verb is in fact the object of an ellipted preposition, rather than the object of the intransitive verb. As for Example 5d, its complete meaning is “昨天小明已经邀请了老师，我今天不邀请**老师/他 (the teacher/him)** 了” (J. Wang, 2001; X. Wang, 2023; X. Wang & Zhou, 2023; Xu, 2001). In these constructions, the object of the transitive verb is only phonetically omitted by virtue of the specific context, while the ellipted object will be retrieved and supplemented. These studies also indicate that the ellipsis of prepositions and objects is motivated by the pragmatic principle of economy. However, pragmatic factors can only affect the surface phonetic form of a specific construction. The factor that determines the actual transitivity features of a given construction lies in the inherent transitivity properties of the verb. Therefore, just like English and other languages, Chinese does have a clear demarcation between intransitive and transitive verbs.

It should be noted that intransitive verbs can be further divided into unaccusative verbs and unergative verbs based on differences in argument mapping mechanisms. Specifically, the distinctions between unaccusative verbs and transitive verbs are reflected not only in transitivity features (intransitive vs. transitive) but also in argument mapping patterns (non-canonical mapping vs. canonical mapping, see Kemmerer (2025), Perlmutter (1978) and other relevant studies for detailed analyses). Considering the particularity of unaccusative verbs, X. Wang (2023) only investigated the processing of Chinese unergative verbs (a subclass of intransitive verbs) and transitive verbs through behavioral experiments. The results showed that native Chinese speakers exhibited longer reaction times and lower accuracy rates when processing transitive verbs. This study also pointed out that future research should pay attention to the neural activities underlying the processing of Chinese intransitive and transitive verbs, so as to form a mutual correspondence between behavioral responses and processing-related neural activities. The present study intends to adopt functional magnetic resonance imaging (fMRI) to compare the neural activities of Chinese intransitive and transitive verbs processing. Meanwhile, E-Prime software will be used to synchronously record the reaction time and accuracy rate of processing the above two types of verbs. From both behavioral and neural perspectives, the present study aims to provide more comprehensive empirical references for analyzing the theoretical controversy over the intransitive-transitive verb dichotomy in Chinese.

## 2. Materials and Methods

### 2.1. Participants

A total of 25 native Chinese-speaking college students or postgraduates were recruited for the present study. Among them, 12 were male participants and 13 were female participants, with ages ranging from 18 to 25 years old. All participants completed the Edinburgh Handedness Inventory and were confirmed to be right-handed, with normal or corrected-to-normal visual acuity. None of them had a history of traumatic brain injury, neurological disorders, or genetic diseases. All participants voluntarily took part in our experiment. Prior to the experiment, they signed the informed consent form and experimental agreement and filled in relevant information. We informed the participants of the precautions for the fMRI laboratory and provided them with training related to the magnetic resonance experiment before the formal test. Appropriate remuneration was given to the participants upon completion of the experiment.

### 2.2. Stimuli

A total of 21 Chinese intransitive verbs and 21 transitive verbs were selected as the target stimuli in our experiment, with each category consisting of 8 monosyllabic verbs and 13 disyllabic verbs. The 21 Chinese intransitive verbs selected for this experiment are as follows: “睡” (to sleep), “笑” (to laugh), “飞” (to fly), “闹” (to act up), “吐” (to vomit), “乐” (to brighten up), “醒” (to wake up), “哭” (to cry), “休息” (to rest), “倒退” (to back up), “升高” (to rise), “开工” (to start work), “工作” (to work), “降落” (to touch down), “下降” (to descend), “前进” (to advance), “咳嗽” (to cough), “后退” (to retreat), “结婚” (to marry), “撤退” (to withdraw), “生气” (to get angry). The corresponding 21 Chinese transitive verbs are: “洗” (to wash), “烤” (to roast), “煮” (to boil), “买” (to buy), “晒” (to air), “卖” (to sell), “抓” (to grasp), “喂” (to feed), “隔离” (to isolate), “处理” (to handle), “清理” (to clean up), “审问” (to interrogate), “邀请” (to invite), “逮捕” (to arrest), “开除” (to expel), “通知” (to inform), “调整” (to adjust), “接收” (to receive), “检查” (to inspect), “修理” (to repair), “救治” (to treat).

To eliminate the confounding effect of the difference in the argument mapping pattern, only unergative verbs were included as the intransitive verb in the present experiment, meaning that the only potential difference between the selected intransitive and transitive verbs lies in their transitivity features. When selecting Chinese transitive verbs, we followed the criteria proposed by Xu (2001) as well as W. Wang and Chen (2017), namely that a verb is identified as transitive if it can be embedded in both passive constructions and bǎ-constructions. For the selection of Chinese unergative verbs, we adopted the method used by X. Wang (2023)and X. Wang et al. (2021, 2023). First, intransitive verbs were filtered out based on the criterion that they cannot be used in passive constructions and bǎ-constructions; subsequently, unergative verbs were further screened from these intransitive verbs according to the standard that they can only freely occur in the NP-V word order.

Malyutina and den Ouden (2017) pointed out that the lexical decision task is likely unable to effectively detect the lexical information encoded in verbs. X. Wang (2023), in turn, argued that this task may largely tap into the orthography of Chinese characters rather than the lexical information of Chinese words. Drawing on the methodology of X. Wang (2023), our study embedded Chinese intransitive and transitive verbs in quantitative constructions [e.g.,两三回 (two or three times)] to effectively probe potential differences in transitivity features reflected in the lexical information of these two verb types. Specifically, each of the 21 intransitive verbs and 21 transitive verbs included in the experiment was used twice, paired with different quantitative constructions respectively. As a result, the experiment contained 42 instances of intransitive-verb quantitative constructions (henceforth intr-quantitative construction) and 42 instances of transitive-verb quantitative constructions (henceforth tr-quantitative construction), with both categories comprising 36 semantically plausible constructions and 6 semantically implausible ones. To effectively investigate whether there is a distinction between transitive and intransitive features at the lexical information level of Chinese verbs, neither subjects nor objects were present in the tested structures. The surface structure of both types of verb quantitative constructions takes the form of V + quantitative construction. In addition to the two types of target structures mentioned above, the experiment also included 42 filler corpus stimuli. Examples of the corpus stimuli are presented in Table 1.

In the present experiment, the semantic anomaly structures were incorporated to prompt participants to perform semantic plausibility judgment tasks during the experiment, thereby compelling them to conduct in-depth processing of each verb. Nevertheless, semantic anomaly structures are not representative corpus stimuli in natural language. To eliminate the potential interference of such structures on the experimental results, only the 36 semantically plausible structures under each verb type were included for analysis in the subsequent statistical processing of behavioral data and fMRI data. Following the procedure of X. Wang (2023) and X. Wang et al. (2021), we calculated and compared the word frequencies of Chinese intransitive and transitive verbs embedded in semantically plausible structures, based on the Chinese Word Frequency List developed by Cai and Brysbaert (2010). The mean and standard deviation of word frequency for intransitive verbs were 2.93 ± 0.52, while those for transitive verbs were 2.90 ± 0.51. Results of the paired-samples *t*-test indicated no significant difference in word frequency between the two verb types [*t*(35) = 0.26, *p* = 0.78, Cohen’s d = 0.46, 95% CI (−3.73, 0.38)]. We also computed and compared the stroke counts of Chinese intransitive and transitive verbs within semantically plausible structures. The mean and standard deviation of stroke count for intransitive verbs were 13.72 ± 5.52, whereas those for transitive verbs were 15.67 ± 6.22. The paired-samples *t*-test revealed no significant difference in stroke count between the two verb categories either [*t*(35) = 1.56, *p* = 0.13, Cohen’s d = 0.26, 95% CI (−0.74, 0.59)]. Among the 36 intransitive and 36 transitive verbs embedded respectively in the two types of semantically plausible structures, each verb category included 13 monosyllabic and 23 disyllabic tokens. All post-verbal quantitative constructions were uniformly trisyllabic. Consequently, the syllable lengths of semantically plausible intr-quantitative constructions and those of tr-quantitative constructions were well-matched: for each construction type, there were 13 four-syllable items and 23 five-syllable items.

Based on a 5-point Likert scale (1 = completely unacceptable, 2 = relatively unacceptable, 3 = uncertain, 4 = relatively acceptable, 5 = completely acceptable), 30 college students or postgraduates who did not participate in the formal experiment rated the rationality of the two syntactic structures included in the present study. Results of paired-samples *t*-tests indicated that for semantically plausible structures, the mean scores and standard deviations of the quantity constructions with Chinese intransitive verbs and transitive verbs were 4.91 ± 0.03 and 4.90 ± 0.04, respectively, with no significant difference between the two structures [*t*(29) = 0.94, *p* = 0.35, Cohen’s d = 0.17, 95% CI (−0.19, 0.53)]. For semantically implausible structures, the mean scores and standard deviations of the corresponding two constructions were 1.05 ± 0.10 and 1.07 ± 0.10, respectively, and no significant difference was observed between them either [*t*(29) = 1.00, *p* = 0.33, Cohen’s d = 0.18, 95% CI (−0.54, 0.18)].

### 2.3. Design

Drawing on the fMRI studies conducted by Feng et al. (2020), X. Wang (2023) and X. Wang et al. (2021) which investigated the neural mechanisms underlying Chinese verb processing, the present study adopted a block design to extract blood oxygen level-dependent (BOLD) signals during the experiment. As indicated in the aforementioned studies, contrasting brain activity between linguistic task conditions and non-linguistic baseline conditions can effectively detect the brain regions that are recruited when participants engage in language processing. Accordingly, the current experiment incorporated not only linguistic tasks but also non-linguistic baseline tasks. The experiment was divided into 3 fMRI scanning sessions, with each session comprising 8 blocks. Each block contained 7 trials: for linguistic task blocks, 6 trials were semantically congruent structures, whereas 1 trial was a semantically incongruent structure; for baseline task blocks, 6 trials displayed leftward arrows, and 1 trial presented a rightward arrow. All stimulus types were included in each of the 3 fMRI scanning sessions. The three linguistic stimulus types—Chinese intr-quantitative construction, tr-quantitative construction, and filler materials—were presented in a Latin square design across the 3 fMRI scanning sessions to counterbalance order effects. The baseline task was administered at the end of each fMRI scanning session.

**Figure 1:**
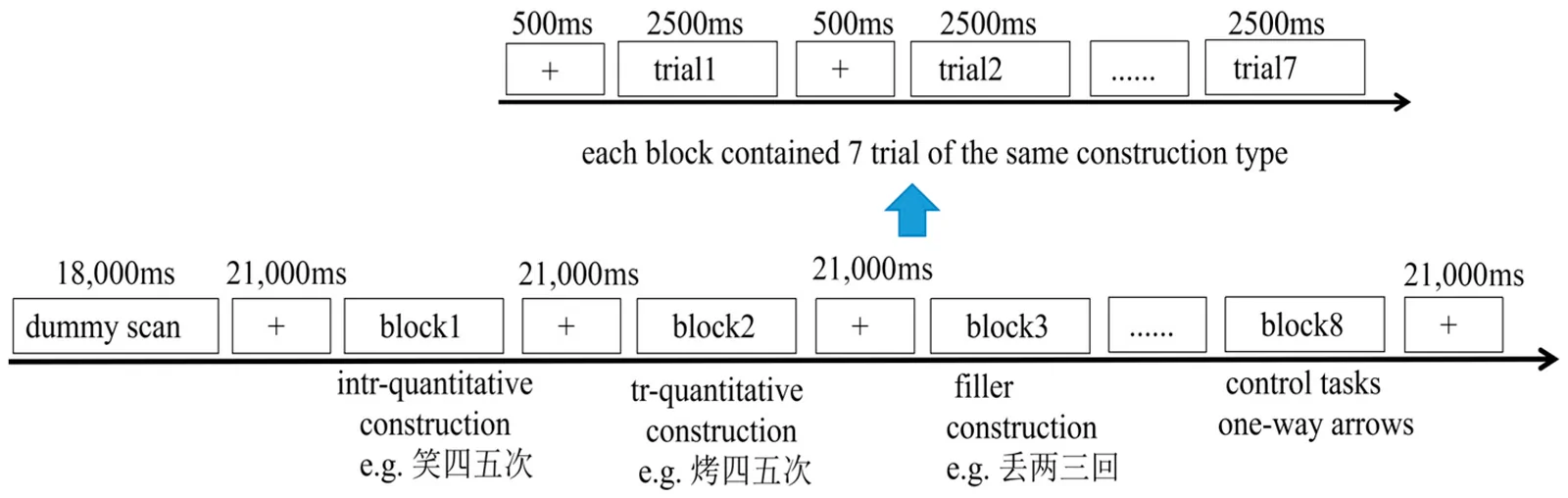
Example of one fMRI scanning session in the present experiment. The English translation of the Chinese corpus in the figure is shown in Table 1.

As illustrated in Figure 1, each trial was presented for 2500 ms, preceded by a 500 ms fixation period during which a central cross “+” was displayed on the screen. Each block lasted 21,000 ms and was preceded by a 21,000 ms inter-block interval (IBI), with the fixation cross “+” remaining on the screen throughout the rest period. A 21,000 ms rest period was also added after the final block of each fMRI scanning session, with the fixation cross still presented. Prior to the commencement of each fMRI scanning session, an 18,000 ms dummy scan was implemented to eliminate T1 saturation effects. Based on these parameters, the total duration of each task block was calculated to be 375 s. During the experiment, after the presentation of stimulus materials, participants were required to make keypress responses as quickly as possible based on semantic plausibility and arrow direction: the left key was pressed for semantically plausible structures paired with left-pointing arrows, while the right key was pressed for semantically implausible structures paired with right-pointing arrows. To ensure that participants were familiar with the experimental task and procedure, a practice session was conducted prior to the formal experiment, and none of the linguistic materials used in the formal experiment appeared in the practice session.

### 2.4. Data Acquisition

Brain imaging data were collected using an MR750 3.0T magnetic resonance imaging (MRI) scanner at the Jiangsu Key Laboratory of Language and Cognitive Neuroscience, Jiangsu Normal University. Functional images were acquired with an echo-planar imaging-gradient echo (EPI-GRE) sequence, with the following parameters: repetition time (TR) = 2000 ms, echo time (TE) = 35 ms, flip angle = 90°, voxel size = 3.5 × 3.5 × 3.5 mm^3^, pixel matrix = 64 × 64, field of view (FOV) = 224 mm^2^, and number of scanning slices = 32. Structural images were obtained using a three-dimensional magnetization-prepared rapid gradient-echo (3D-MPRAGE) sequence, with the parameters as follows: TR = 8200 ms, TE = 3.2 ms, flip angle = 12°, voxel size = 1.0 × 1.0 × 1.0 mm^3^, pixel matrix = 256 × 256, FOV = 240 mm^2^, and number of scanning slices = 32. Behavioral data, including accuracy rate and reaction time, were recorded using E-Prime software.

### 2.5. Data Analysis

As mentioned earlier, only semantically plausible constructions were included in the processing of behavioral data and fMRI data. Paired-samples *t*-tests were conducted in SPSS Statistics Version 19.0 to compare performance differences between intr-quantitative constructions and tr-quantitative constructions, with accuracy and reaction time (RT) as the dependent measures. For RT analyses, only data from correct trials were included to exclude potential confounding effects of response errors.

On a whole-brain scale, functional magnetic resonance imaging (fMRI) data were processed using the Statistical Parametric Mapping 12 version (SPM12) software package (http://www.fil.ion.ucl.ac.uk/spm/software/spm12/, accessed on 1 July 2019) running on MATLAB R2010 (MathWorks, Natick, MA, USA). Preprocessing steps were implemented as follows: first, the initial 9 dummy scans (acquired within the first 18 s of the scanning session) were discarded. The remaining functional images were then realigned to the mean functional image to minimize artifacts induced by head motion, followed by coregistration of functional images to the corresponding T1-weighted structural image for each participant. Next, spatial normalization was performed to warp all images into the standard Montreal Neurological Institute (MNI) space, and tissue segmentation was carried out to partition the brain into three tissue types: gray matter, white matter, and cerebrospinal fluid (CSF). Finally, functional images were spatially smoothed with an isotropic Gaussian kernel of 7 mm full-width at half-maximum (FWHM) to improve the signal-to-noise ratio (SNR) and conform to the assumptions of Gaussian random field theory. For first-level (individual) statistical analyses, paired-samples *t*-tests were conducted to contrast the neural activation patterns associated with two pairwise comparisons: (1) intr-quantitative constructions versus tr-quantitative constructions. (2) each target linguistic condition versus the baseline condition. Subsequently, second-level (group) statistical analyses were performed to aggregate activation maps across all participants for each comparison condition.

Drawing on the fMRI studies of Chinese language processing conducted by Feng et al. (2015, 2020), X. Wang (2023) and X. Wang et al. (2021), after the whole-brain analysis, we conducted a region-of-interest (ROI) analysis on the fMRI data in light of the research questions and objectives. Previous studies (Den Ouden et al., 2009; Meltzer-Asscher et al., 2015; Thompson et al., 2007, 2013) have demonstrated that, compared with intransitive verbs, transitive verbs elicit significantly stronger activations in several functional brain regions, including the bilateral angular gyrus (BA 39), the left supramarginal gyrus (BA 40) and the left inferior frontal gyrus (BA 44/45). These brain regions are identified as the core areas underlying the neural correlates of the transitivity features of verbs. On the basis of these regions, we further carried out separate ROI analyses for the intr-quantitative construction and the tr-quantitative construction. With reference to Feng et al. (2015, 2020) and X. Wang et al. (2021), the regions of interest (ROIs) comprised 5 mm spheres determined from the peak activational areas of the comparison between intr-quantitative constructions and tr-quantitative constructions. These areas included the peak activation cluster in the left angular gyrus (MNI coordinates: −31, −56, 42; BA39), that in the right angular gyrus (MNI coordinates: 36, −49, 39; BA 39), that in the left supramarginal gyrus (MNI coordinate −48, −39, 35; BA40) and that in the left inferior frontal gyrus (MNI coordinate −41, 25, 18; BA44). We employed the Marsbar analysis toolbox, which is compatible with SPM12, to separately extract the signal changes of intr-quantitative constructions and tr-quantitative constructions in the aforementioned 4 ROIs. We further performed paired-samples *t*-tests on the signal changes of the two constructions across these 4 ROIs.

## 3. Results

### 3.1. Behavioral Results

**Figure 2:**
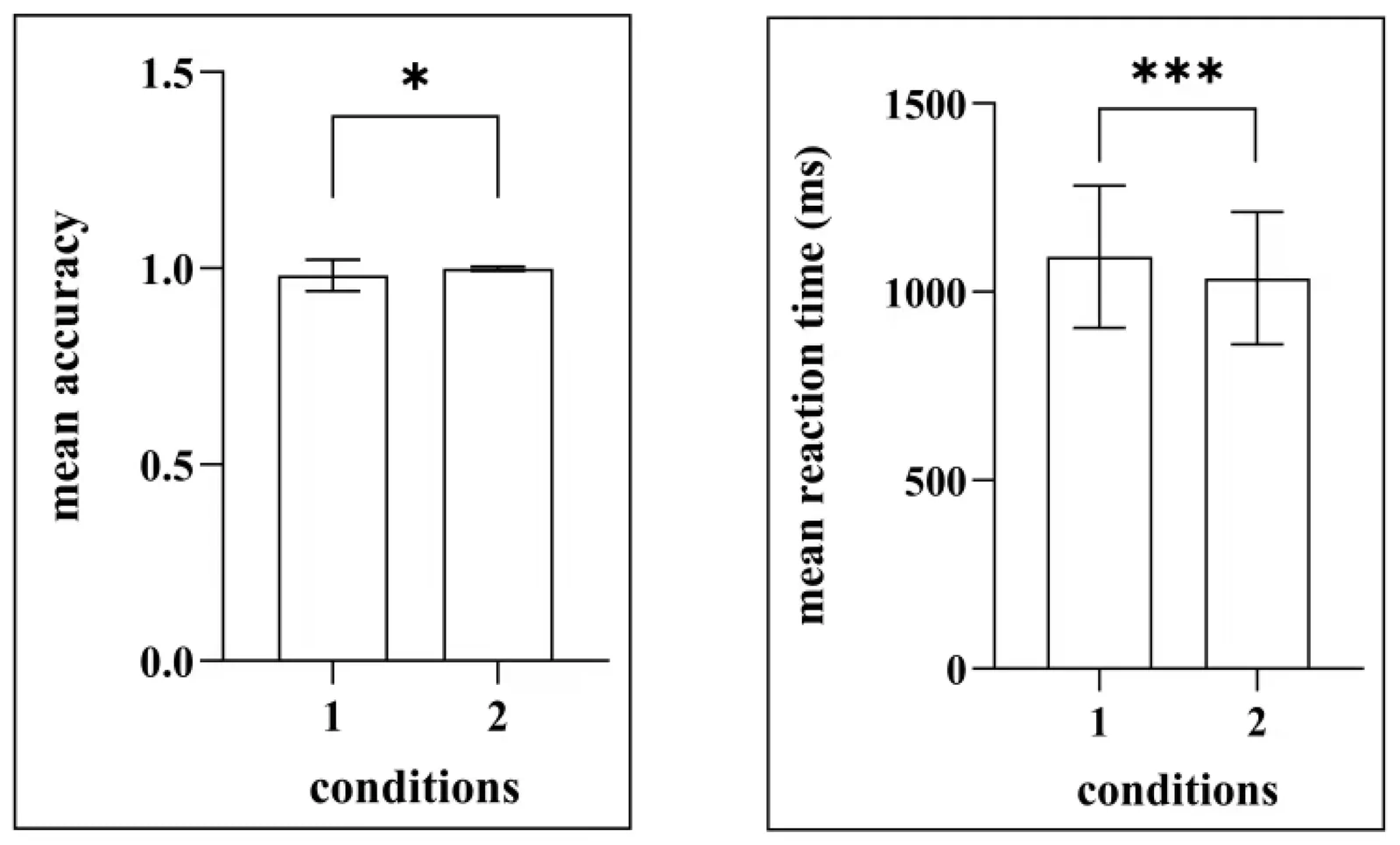
Comparison of accuracy rate and reaction time between intr-quantitative and tr-quantitative constructions. 1 = tr-quantitative constructions; 2 = intr-quantitative constructions. “*” denotes *p* < 0.05; “***” denotes *p* < 0.001.

**Figure 3:**
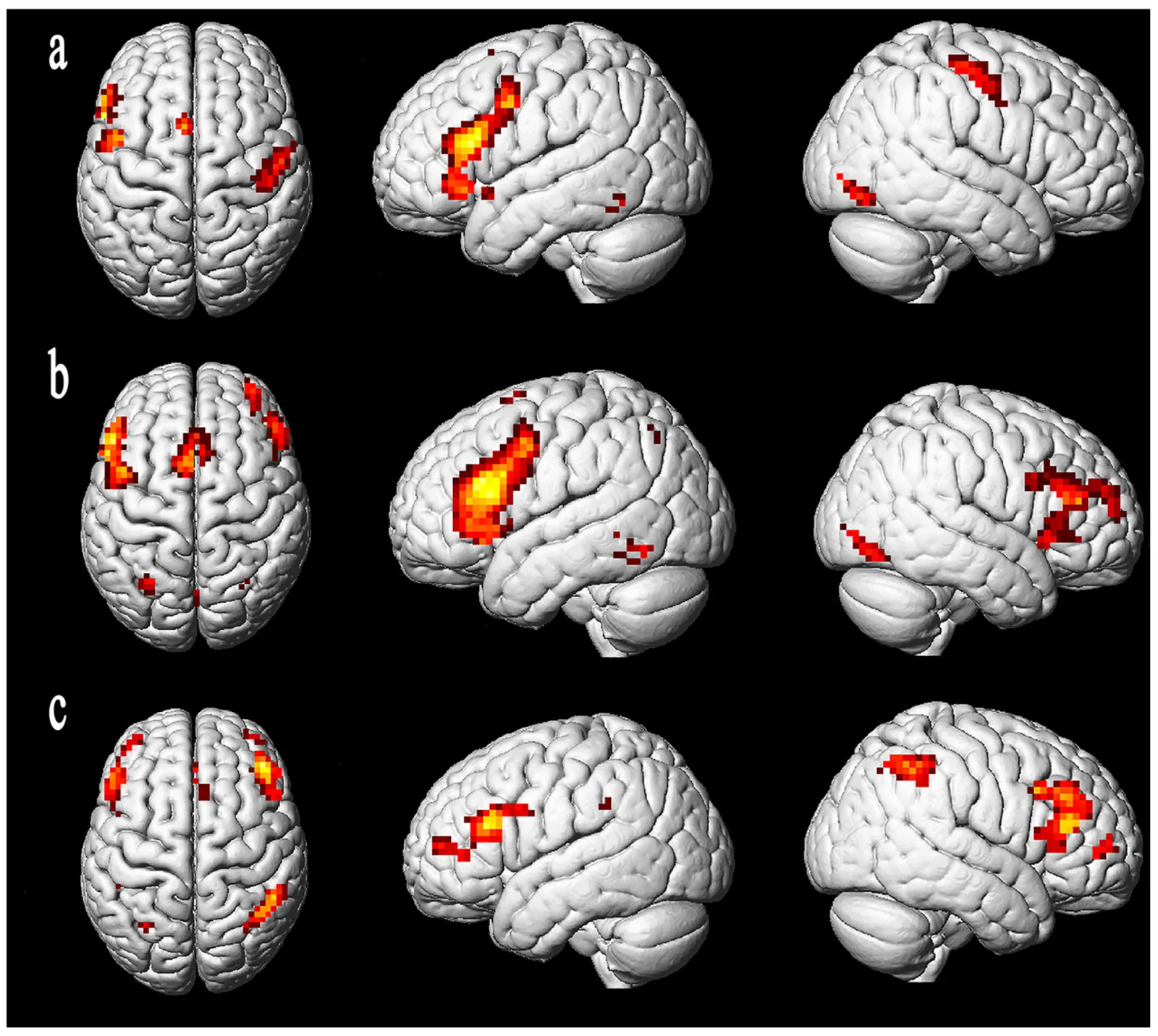
Regions of differential activation for different comparison conditions (FDR correction, *p* < 0.05). Notes: (**a**) intr-quantitative constructions > baseline; (**b**) tr-quantitative constructions > baseline; (**c**) tr-quantitative constructions > intr-quantitative constructions. The color intensity in the figure indicates the strength of brain activation, with the exact T-values for each region provided in Table 2.

The results of the paired-samples *t*-tests showed that the mean accuracy with standard deviation for the intr-quantitative constructions was 0.999 ± 0.006, while that for the tr-quantitative constructions was 0.982 ± 0.04. A significant difference in accuracy was observed between the two constructions [*t*(24) = 2.24, *p* < 0.05, Cohen’s d = 0.45, 95% CI (0.03, 0.86)], with the accuracy of the intr-quantitative constructions being significantly higher than that of the tr-quantitative constructions (see Figure 2). Meanwhile, the mean reaction time with standard deviation for the intr-quantitative constructions was 1035.79 ± 175.71 ms, compared to 1093.36 ± 189.09 ms for the tr-quantitative constructions. A significant difference was also found in reaction time between the two constructions [*t*(24) = −5.30, *p* < 0.001, Cohen’s d = −1.06, 95% CI (−1.54, −0.56)], such that the reaction time for the intr-quantitative constructions was significantly shorter than that for the tr-quantitative constructions (see Figure 3). We further compared the accuracy rate and reaction time between the linguistic task and the non-linguistic task (baseline task). For the accuracy rate, the linguistic task yielded a mean value of 0.991 ± 0.02, whereas the non-linguistic task showed a higher mean accuracy of 0.999 ± 0.004. In terms of reaction time, the linguistic task resulted in a longer mean reaction time of 1064.58 ± 180.49 ms, while the non-linguistic task had a shorter mean reaction time of 618.76 ± 79.67 ms. Paired-samples *t*-tests revealed significant differences between the two tasks both in accuracy rate [*t*(24) = −2.27, *p* < 0.05, Cohen’s d = −0.45, 95% CI (−0.86, −0.04)] and reaction time [*t*(24) = 15.15, *p* < 0.001, Cohen’s d = −3.03, 95% CI (−3.96, −2.08)].

### 3.2. fMRI Results

The results of whole-brain analysis are as follows: compared with the non-linguistic baseline task, the intr-quantitative construction elicited significant activation in the left middle frontal gyrus, left inferior frontal gyrus, left fusiform gyrus, left superior frontal gyrus, right middle frontal gyrus, right postcentral gyrus, right occipital lobe, and right fusiform gyrus (see Table 2 and a condition in Figure 3); Compared with the non-linguistic baseline task, the tr-quantitative construction elicited significant activation in multiple brain regions, including the left inferior frontal gyrus, left middle frontal gyrus, left medial middle frontal gyrus, left superior frontal gyrus, left inferior parietal lobule, left fusiform gyrus, left lingual gyrus, right cingulate gyrus, right occipital lobe, right fusiform gyrus, right inferior frontal gyrus, and right angular gyrus (see Table 2 and b condition in Figure 3); Compared with the intr-quantitative construction, the tr-quantitative construction also elicited significant activation in multiple brain regions, including the left inferior frontal gyrus (LIFG), left angular gyrus (LAG), left inferior parietal lobule, left supramarginal gyrus (LSG), right middle frontal gyrus, right inferior frontal gyrus, right angular gyrus (RAG), right inferior parietal lobule, right superior parietal lobule, and right medial middle frontal gyrus (see Table 2 and condition (c) in Figure 3). Additionally, no functionally significant brain regions were activated in non-linguistic tasks compared with linguistic tasks, as well as in intr-quantitative constructions compared with tr-quantitative constructions.

**Figure 4:**
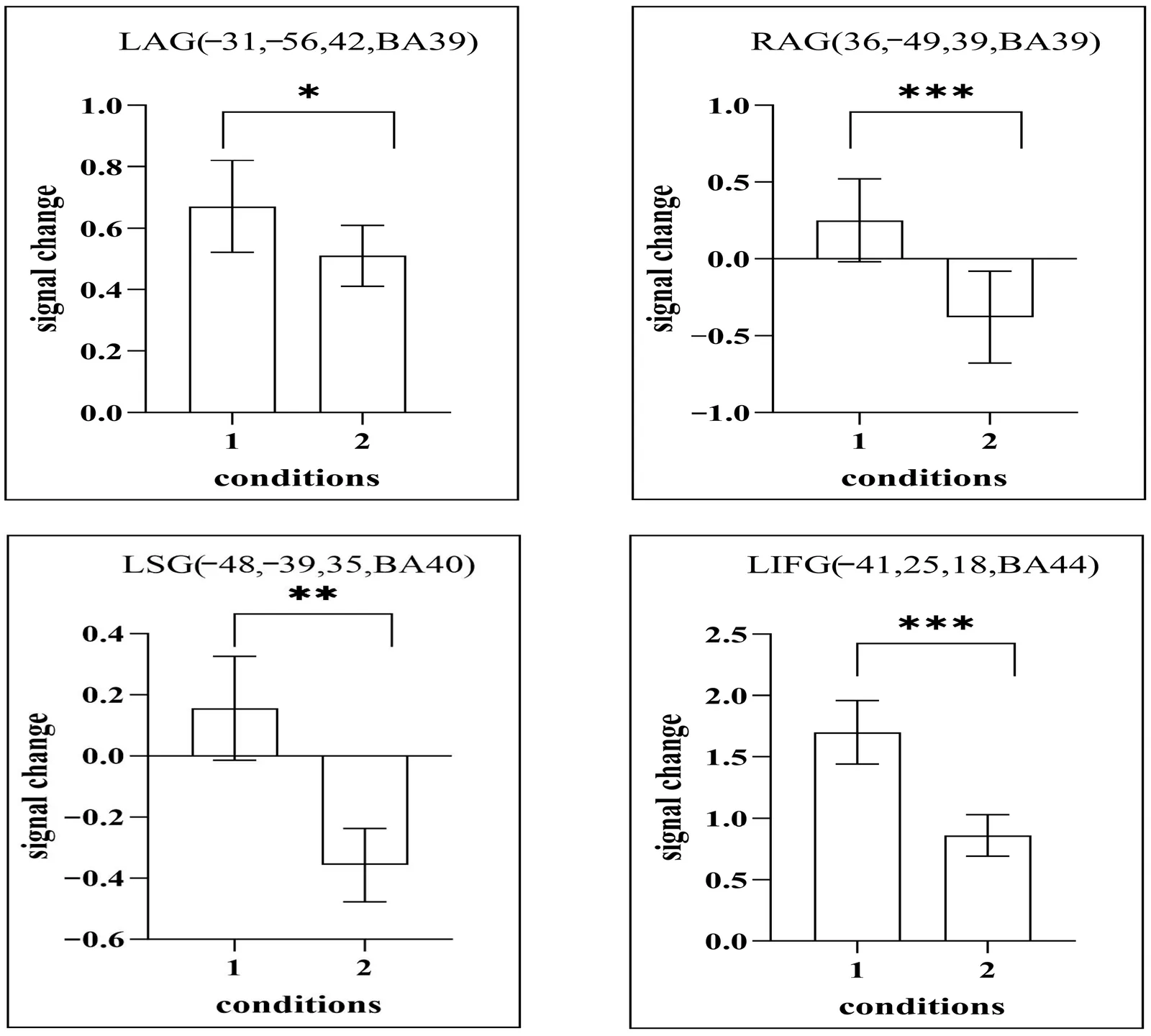
Signal changes of the Chinese intr-quantitative constructions and tr-quantitative constructions, extracted by ROI analysis on the left angular gyrus/LAG [peak location was at the point (−31, −56, 42), BA39], right angular gyrus/RAG [peak location was at the point (36, −49, 39), BA39], left supramarginal gyrus/LSG [peak location was at the point (−48, −39, 35), BA40], and left inferior frontal gyrus/LIFG [peak location was at the point (−41, 25, 18), BA44]. 1 = tr-quantitative constructions > baseline; 2 = intr-quantitative constructions > baseline. “*” denotes *p* < 0.05, “**” denotes *p* < 0.01, and “***” denotes *p* < 0.001.

As illustrated in Figure 4, the results of ROI analysis are as follows: the activation elicited by tr-quantitative constructions was significantly stronger than that elicited by intr-quantitative constructions in four regions of interest (ROIs)—the left angular gyrus, right angular gyrus, left supramarginal gyrus, and left inferior frontal gyrus. The results of paired-samples *t*-tests were as follows: the left angular gyrus (BA39): *t*(24) = 2.40, *p* < 0.05, Cohen’s d = 0.48, 95% CI (0.06, 0.89); the right angular gyrus (BA39): *t*(24) = 4.42, *p* < 0.001, Cohen’s d = 0.88, 95% CI (0.41, 1.34); the left supramarginal gyrus (BA40): *t*(24) = 3.52, *p* < 0.01, Cohen’s d = 0.70, 95% CI (0.26, 1.73); and the left inferior frontal gyrus (BA44): *t*(24) = 4.12, *p* < 0.001, Cohen’s d = 0.84, 95% CI (0.37, 1.29).

## 4. Discussion

### 4.1. Discussion of Behavioral Results

The two critical behavioral indices, accuracy rate and reaction time, are generally regarded as reflections of language processing complexity. Specifically, higher complexity in linguistic operations (e.g., syntactic and semantic processing) correlates with lower accuracy and longer reaction times. A substantial body of research has demonstrated that verbs with a greater number of arguments (higher transitivity complexity) are associated with decreased processing accuracy and increased reaction latency (Ahrens, 2003; Ahrens & Swinney, 1995; Den Ouden et al., 2009; Kim & Thompson, 2000, 2004; Thompson et al., 1997, 2010, 2013). Among these studies, several have specifically focused on comparing the processing of intransitive verbs and transitive verbs.

For instance, Thompson et al. (2010) employed a lexical decision task to examine the processing of intransitive and transitive verbs among 10 normal participants. Statistical results revealed that the average reaction time for transitive verb processing was longer than that for intransitive verb processing, with a marginally significant difference between the two. Kim and Thompson (2004) adopted a lexical naming task to investigate verb processing in 14 patients with Alzheimer’s disease and 9 patients with grammatical aphasia, and found that Alzheimer’s disease patients achieved significantly higher accuracy rates in naming intransitive verbs than transitive verbs. Patients with grammatical aphasia exhibited a similar pattern of difference in the naming of these two verb types, namely, a higher accuracy rate in processing intransitive verbs. Two earlier studies on aphasia demonstrated that, both in lexical naming tasks and verb categorizing tasks, aphasic populations showed lower accuracy in processing transitive verbs than intransitive verbs (Kim & Thompson, 2000; Thompson et al., 1997).

**Figure 5:**
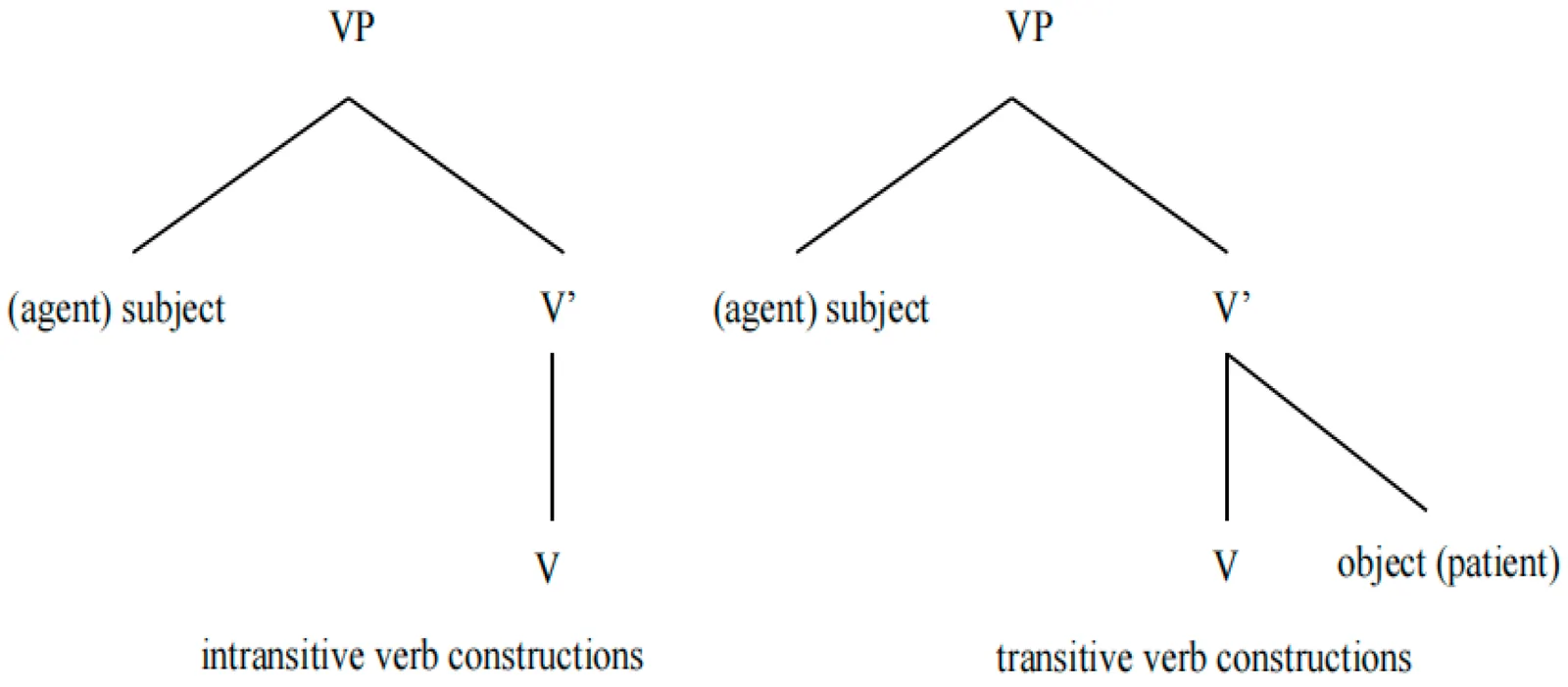
Syntactic and semantic feature comparisons between intransitive and transitive verbs.

When analyzing the differences in accuracy rates and reaction times between intransitive and transitive verbs, the aforementioned studies indicated that, as illustrated in Figure 5, the lexical information of intransitive verbs dictates that such verbs involve only a subject at the syntactic level and solely the semantic representation of the subject at the semantic level. In contrast, the lexical information of transitive verbs specifies that these verbs entail the syntactic representation of two constituents (the subject and the object) and the corresponding semantic representations of both elements. The discrepancy in complexity between intransitive and transitive verbs in terms of their syntactic and semantic representations renders the former more amenable to processing and the latter more computationally demanding. Such differences in processing difficulty are likely manifested as variations in the accuracy rate and the reaction time at the behavioral level. Therefore, lower accuracy and longer reaction time may well reflect the more intricate syntactic and semantic processing required for transitive verbs relative to intransitive counterparts.

Turning to the present study, consistent with the aforementioned behavioral experiments investigating the processing of intransitive and transitive verbs, our findings demonstrated that in structures where neither the subject nor the object was overtly expressed, Chinese transitive verbs elicited significantly lower accuracy and longer reaction times than intransitive verbs. Drawing on the interpretations of behavioral differences (accuracy and reaction time) between intransitive and transitive verb processing proposed in previous research, we argue that the reduced accuracy and prolonged reaction time observed for Chinese tr-quantitative constructions, as opposed to intr-quantitative ones, reveal that transitive verbs encode more complex syntactic and semantic information than their intransitive counterparts. Specifically, by virtue of the verb’s inherent lexical information, Chinese intr-quantitative constructions activate the propositional content structured as “X + V + quantitative phrase”. For instance, the expression “笑四五次/laugh four-five times” activates the conceptualization of *someone laughed four or five times*. Correspondingly, Chinese tr-quantitative constructions, via the verb’s intrinsic lexical specifications, activate the propositional schema of “X + V + quantitative phrase + Y”. For example, the expression “烤四五次/roast four-five times” activates the conceptual structure of *someone roasted something four or five times*. Evidently, compared with the schema “X + V + quantitative phrase”, the structure “X + V + quantitative phrase + Y” involves a greater number of syntactic constituents (adding the object constituent) and a more sophisticated semantic representation (incorporating the semantic features of the object). Thus, the behavioral differences in accuracy and reaction time between Chinese intr-quantitative and tr-quantitative constructions are likely attributable to the divergent lexical information encoded by intransitive and transitive verbs. The analysis here further suggests that, based on the disparities in behavioral data (accuracy rate and reaction time), Chinese verbs can be dichotomized into intransitive and transitive categories.

### 4.2. Discussion of fMRI Results

First, whole-brain analysis revealed that relative to the non-linguistic task, the two linguistic-task conditions elicited significant activation in the left frontal, temporal, and parietal lobes. This finding aligns with well-established evidence that implicates these left-lateralized regions as core substrates for language processing (Friederici, 2017; Kochi et al., 2023; Krieger-Redwood et al., 2024; Pei et al., 2023; Solomons et al., 2024; L. Wang et al., 2025; Yan et al., 2025; Zeng et al., 2025; Zhao et al., 2024). Furthermore, it corroborates the methodological validity of identifying linguistic structure-specific activation patterns by contrasting linguistic conditions against the baseline. Critically, direct comparisons indicated that Chinese tr-quantitative constructions elicited greater activation in the bilateral frontal and parietal lobes, including the supramarginal gyrus and the angular gyrus, compared with Chinese intr-quantitative constructions. These results suggest that Chinese tr-quantitative constructions impose substantially higher neural processing demands than Chinese intr-quantitative constructions.

Moreover, fMRI studies focusing on transitive and intransitive verb processing in languages other than Chinese have reported that transitive verb processing induces enhanced activation in the left angular gyrus (BA39), the left supramarginal gyrus (BA40), and the left inferior frontal gyrus (BA44/45), relative to intransitive verb processing. For example, Thompson et al. (2007) examined and compared brain activation patterns in 14 normal participants during the naming of intransitive verbs, transitive verbs, and ditransitive verbs. Experimental results of this study indicated that, compared with intransitive verbs, the processing of transitive verbs elicited stronger activation in brain regions such as the left angular gyrus (BA39) and the left supramarginal gyrus (BA40). Another fMRI study, which also investigated and compared neural activity associated with the processing of intransitive and transitive verbs under a lexical naming task (Den Ouden et al., 2009), found that compared with intransitive verbs, the processing of transitive verbs elicited stronger activation not only in the bilateral angular gyri (BA39) and supramarginal gyri (BA40), but also in the left inferior frontal gyrus (BA44/45). In addition, the fMRI study (Thompson et al., 2013) investigating aphasic populations revealed that, compared with intransitive verbs, the processing of transitive verbs in patients with grammatical aphasia triggered stronger activation in brain regions such as the bilateral angular gyri and supramarginal gyri.

The bilateral angular gyri (BA39) and the left supramarginal gyrus (BA40) are closely associated with the retrieval, selection, and integration of lexical semantics (Bao & Xuan, 2025; Kemeny et al., 2006; Vigneau et al., 2006; Zeng et al., 2025; Y. Zhang et al., 2024). Concurrently, theoretical analyses posit that the lexical representation of transitive verbs encodes two semantic roles, corresponding respectively to the syntactic functions of subject and object, whereas intransitive verbs only encode the semantic role linked to the subject. This suggests that the semantic integration of lexical information for transitive verbs, primarily the retrieval of semantic roles associated with potential subjects and objects, is more complex than that for intransitive verbs. Therefore, enhanced activation in the bilateral angular gyri and left supramarginal gyrus reflects the more elaborate semantic representation processes inherent to transitive verbs relative to intransitive counterparts (Den Ouden et al., 2009; Meltzer-Asscher et al., 2015; Thompson et al., 2007, 2013).

These conclusions appear applicable to the findings of the present study. As noted earlier in our analysis of the behavioral results, due to differences in transitivity features at the lexical level, Chinese tr-quantitative constructions entail a more sophisticated semantic representation, incorporating the semantic role of a potential object. Accordingly, the enhanced activation observed in the bilateral angular gyri and left supramarginal gyrus in our experiment most likely reflects the more complex argument–semantic representation process of transitive verbs compared to intransitive verbs at the semantic level. This finding further corroborates the existence of a binary distinction between transitive and intransitive verbs at the lexical level in Chinese.

In addition, the left inferior frontal gyrus, particularly BA44, is generally regarded as closely associated with syntactic processing (Friederici, 2017). Relevant studies have found that complex syntactic processing tends to induce stronger activation in the left inferior frontal gyrus (BA44) than simple syntactic processing (Jeong et al., 2025; Sun et al., 2021; Yang et al., 2024). Meanwhile, theoretical analyses suggest that the initial syntactic structure encoded in the lexical information of transitive verbs is more complex than that of intransitive verbs: the former involves two merging operations (for the subject and the object), while the latter only involves one merging operation (for the subject). When investigating the neural mechanisms underlying verb argument structure processing, Den Ouden et al. (2009), as well as Thompson and Meltzer-Asscher (2014), pointed out that the left inferior frontal gyrus (BA44) is likely to reflect the more complex initial syntactic processing of transitive verbs compared to intransitive verbs.

Interestingly, the analyses of the left inferior frontal gyrus presented by Den Ouden et al. (2009) and Thompson and Meltzer-Asscher (2014) also seem applicable to explaining the results of the present experiment. Specifically, Chinese intr-quantitative constructions only involve the syntactic representation of the subject. In contrast, Chinese tr-quantitative constructions involve not only the representation of the subject but also that of the object. Accordingly, the stronger activation in the left inferior frontal gyrus observed in our experiment most likely reflects the more complex initial syntactic construction processing of transitive verbs relative to intransitive verbs. This finding further corroborates the existence of a binary distinction between transitive and intransitive verbs at the lexical level in Chinese.

It should also be noted that our ROI analyses of the bilateral angular gyri, left supramarginal gyrus, and left inferior frontal gyrus are consistent with the model of verb argument structure processing proposed by Thompson and Meltzer-Asscher (2014). According to this model, during the processing of verb argument structure, the semantic information of argument structure is first extracted via the bilateral angular gyri and supramarginal gyrus, followed by the building of the initial syntactic structure of argument structure mediated by the left inferior frontal gyrus.

From a cross-linguistic perspective, our findings indicate that the distinction between intransitive and transitive verbs is likely a universal feature of human language, with processing differences between the two verb types manifested at both behavioral and neural levels. For Chinese specifically, our results reveal that pragmatic factors, including preposition ellipsis and object ellipsis, shape the surface form of verb-object constructions in Chinese, but do not determine their transitivity features, which are inherent to the verbal lexical information.

## 5. Conclusions and Limitations

Transitivity, as a classic topic in linguistics, has attracted extensive attention from academic circles. Theoretical and empirical studies overseas have consistently demonstrated a binary distinction between transitive and intransitive verbs. However, whether such a dichotomy applies to Chinese verbs has long been a subject of theoretical controversy. From an empirical perspective, the present study examines the processing of Chinese transitive and intransitive verbs through the dual lenses of the behavioral response and the corresponding neural activity. Behavioral results revealed that native Chinese speakers exhibited significantly longer reaction time and lower accuracy rate when processing transitive verb constructions with both omitted subjects and objects, compared to intransitive verb constructions with omitted subjects. Neuroimaging findings further indicated that, relative to intransitive structures without subjects, transitive structures without both subjects and objects elicited significantly stronger activation in several regions of interest, including the bilateral angular gyri, the left supramarginal gyrus, and the left inferior frontal gyrus. These results confirm that the behavioral differences observed in processing Chinese transitive and intransitive are underpinned by distinct neural substrates. Based on the experimental findings of our research, we can conclude that the processing difficulty of Chinese transitive and intransitive verbs differs even when verbs appear without subjects or objects, verifying that a binary distinction between intransitive and transitive verbs exists at the lexical level in Chinese.

It should be noted that the present study has some limitations. First, although the present study provides evidence for lexical transitivity differences in Chinese verbs, further research is required to explore whether and how extra-grammatical factors such as context and pragmatics modulate these differences. Future studies could systematically investigate the processing mechanisms of object-omitted constructions with Chinese transitive verbs and noun phrase-following constructions with Chinese intransitive verbs. Second, the sample size of participants is relatively small (but also typical for fMRI studies), which may limit the generalizability of the findings. Third, the semantic judgment task is somewhat artificial, and realistic sentence comprehension tasks may better capture natural processing.

## Figures and Tables

**Table 1:** Sample stimuli of each condition in the present experiment.

Conditions	Semantic Types	Chinese	English
intr-quantitative construction	plausible	笑四五次	laugh four or five times
	implausible	飞十几口	fly more than ten bites
tr-quantitative construction	plausible	烤四五次	roast four or five times
	implausible	晒十多鞭	air more than ten lashes
filler construction	plausible	丢两三回	be lost two or three times
	implausible	死十几句	die more than ten sentences

**Table 2:** Regions of different activation for different comparison conditions.

Conditions	Brain Regions	X	Y	Z	T	*P-*_FDR_	Voxels
intr-quantitative	left middle frontal gyrus (BA6)	−55	0	42	6.51	0.003	417
construction	left inferior frontal gyrus (BA44)	−48	21	18	5.79	0.005	
>	left inferior frontal gyrus (BA45)	−38	25	14	5.55	0.005	
baseline	left fusiform gyrus (BA37)	−38	−53	−11	5.38	0.005	51
	left superior frontal gyrus (BA6)	−6	11	56	4.80	0.008	29
	right middle frontal gyrus	54	−11	53	7.13	0.003	52
	right postcentral gyrus	47	−21	60	6.75	0.003	
	right occipital lobe (BA19)	36	−81	−11	5.61	0.005	33
	right fusiform gyrus (BA18)	29	−84	−4	5.58	0.005	
tr-quantitative	left inferior frontal gyrus (BA44)	−48	25	21	8.13	0.000	770
construction	left inferior frontal gyrus	−45	7	28	7.25	0.000	
>	left middle frontal gyrus (BA6)	−52	4	46	6.72	0.000	
baseline	left medial middle frontal gyrus (BA6)	−3	14	53	7.65	0.001	280
	left superior frontal gyrus (BA6)	−10	7	70	4.47	0.003	
	left inferior parietal lobule (BA7)	−27	−63	42	6.48	0.000	75
	left fusiform gyrus (BA37)	−48	−60	−14	6.44	0.000	76
	left lingual gyrus (BA30)	−6	−70	7	5.01	0.001	117
	right cingulate gyrus (BA32)	5	25	39	6.24	0.000	280
	right occipital lobe (BA19)	40	−74	−14	6.94	0.000	47
	right fusiform gyrus (BA18)	29	−84	−4	6.32	0.000	
	right inferior frontal gyrus	54	35	25	5.38	0.001	288
	right inferior frontal gyrus (BA45)	36	28	11	4,89	0.001	
	right angular gyrus (BA39)	29	−60	39	5.03	0.001	27
tr-quantitative	left inferior frontal gyrus (BA44)	−41	25	18	4.62	0.037	148
constructions	left angular gyrus (BA39)	−31	−56	42	4.20	0.037	46
>	left inferior parietal lobule	−34	−46	35	3.91	0.037	
intr-quantitative	left supramarginal gyrus (BA40)	−48	−39	35	3.91	0.037	
construction	right middle frontal gyrus	40	32	32	5.30	0.037	180
	right inferior frontal gyrus	43	21	35	3.93	0.037	
	right angular gyrus (BA39)	36	−49	39	5.03	0.037	131
	right inferior parietal lobule (BA39)	47	−53	46	4.75	0.037	
	right superior parietal lobule	40	−60	53	4.53	0.037	
	right medial middle frontal gyrus	5	21	49	3.64	0.039	20
	right middle frontal gyrus (BA10)	33	56	4	4.12	0.037	20
	right middle frontal gyrus (BA10)	43	53	7	3.71	0.039	

## Data Availability

The data presented in this study are available on request from the first author and the corresponding author.
